# DORSAL BRAINSTEM SYNDROME AND THE USE OF NEURALLY ADJUSTED VENTILATORY ASSIST (NAVA) IN AN INFANT

**DOI:** 10.1590/1984-0462/;2018;36;1;00003

**Published:** 2017-09-21

**Authors:** José Colleti, Walter Koga, Werther Brunow de Carvalho

**Affiliations:** a Hospital Santa Catarina, São Paulo, SP, Brasil.; b Universidade de São Paulo, São Paulo, SP, Brasil.

**Keywords:** Encephalitis, Critical care, Neurally adjusted ventilatory assist, Encefalite, Terapia intensiva, Suporte ventilatório interativo

## Abstract

**Objective:**

To report a rare case of dorsal brainstem syndrome in an infant after hypoxic-ischemic episode due to severe sepsis and the use of neurally adjusted ventilatory assist (NAVA) to aid in diagnosis and in the removal of mechanical ventilation.

**Case description:**

A 2-month-old male infant, previously healthy, presented with severe sepsis that evolved to dorsal brainstem syndrome, which usually occurs after hypoxic-ischemic injury in neonates and infants, and is related to very specific magnetic resonance images. Due to neurological lesions, thei nfant remained in mechanical ventilation. A NAVA module was installed to keep track of phrenic nerve conduction to the diaphragm, having successfully showed neural conduction and helped removing mechanical ventilation.

**Comments:**

Dorsal brainstem syndrome is a rare condition that should be considered after hypoxic-ischemic episode in infants.

## RESUMO

**Objetivo:**

Relatar um caso raro de síndrome posterior do tronco cerebral em um lactente após um episódio hipóxico-isquêmico devido a sepse grave, e o uso da ventilação assistida ajustada neuralmente no auxílio diagnóstico e no desmame da ventilação mecânica.

**Descrição do caso:**

Lactente masculino de 2 meses de idade, previamente hígido, apresentou sepse grave que evoluiu para síndrome posterior do tronco encefálico, entidade que pode ocorrer após lesão hipóxico-isquêmica em neonatos e lactentes e que apresenta imagens de ressonância magnética muito particulares. Devido à lesão neurológica, permaneceu em ventilação mecânica. Optou-se por iniciar ventilação assistida ajustada neuralmente para verificar a patência da condução do nervo frênico ao diafragma e auxiliar no desmame da ventilação mecânica.

**Comentários:**

A síndrome posterior do tronco cerebral é uma entidade rara que deve ser considerada em lactentes após evento hipóxico-isquêmico.

## INTRODUCTION

Dorsal brainstem syndrome (DBSS) is a rare condition that affects children with hypoxic-ischemic encephalopathy. It may occur after severe sepsis, and Magnetic Resonance (MR) imaging is the method of choice for assessing this kind of injury in neonates and infants.^1^ We report a case of an infant who presented myocardiopathy, and develope DBSS after meningitis-related septic shock. The child became dependent of mechanical ventilation (MV). Neurally adjusted ventilatory assist (NAVA) was successfully used to diagnose the neural conduction through the phrenic nerve, and also helped in MV removal.^2^ ^,^ ^3^

## CASE DESCRIPTION

A two-month-old male infant weighing 6.9 kg was admitted to the pediatric ICU with respiratory distress, tachycardia, hypotonia, cold extremities, and fever. About three days earlier, the infant started with fever (37.8ºC to 38.5ºC), vomiting, apathy and eventually progressed to respiratory distress and cardic arrest. No relevant facts about his previous health history were available.

At admission, the infant was hypotonic, with moderate subdiaphragmatic and intercostal retractions; the skin was pale and cold. Breathing rate was 80 incursions/minute and heart rate was 180 bpm. It was interpreted as circulatory collapse and the infant was put in orotracheal intubation. The nurses tried a peripheral venous access, but dehydration made it difficult. An intraosseous access was achieved. A total of 40 ­mL/­kg of saline solution was infused in 20 minutes. After that, a peripheral venous line was inserted and another 40 ­mL/­kg of saline solution was administered to the patient in 60 minutes. Perfusion was regular and the mean arterial pressure (MAP) was low (30 mmHg). Milrinone (0.5 mcg/kg/min) and norepinephrine (0.2 mcg/kg/min) was started and perfusion improved 30 minutes later. MAP raised to 50 mmHg. Ceftriaxone 100 ­mg/­kg/­day and acyclovir (45 mg/kg/day) were administered in the first hour of admission, as per institutional sepsis protocol. A lumbar puncture was performed to collect cerebrospinal fluid (CSF) and study the hypothesis of central nervous system virus infection.

Peripheral blood count showed 21,780 leucocytes (58% neutrophils, 4% bands, and 54% segmented), 12.6 g/dL hemoglobin, 34.9% hematocrit, and 482,000/mm^3^ platelets. C-protein reaction (CPR) level was 2.48 mg/dL (normal <0.6 mg/dL). Procalcitonin concentration was 7.6 ­ng/­mL (normal <0.5 ng/mL). Lactate level was 50 mg/dL (normal <11.3 mg/dL). Troponin I was 1.67 ng/mL (normal <0.16 ­ng/­mL). Creatine kinase (CK) level was 926 U/L (normal: 38-174 U/L) and creatine kinase MB (CKMB) was 24.1 ng/mL (normal <5 ng/mL). CSF analysis showed white blood cells (WBC) count of 174/mm^3^ (60% polymorphonuclear cells), protein at 100 mg/dL, glucose at 92 mg/dL, and lactate at 33 mg/dL. Diagnosis of viral meningoencephalitis was considered, but CSF cultures for bacteria, fungi, herpes virus, and enterovirus resulted negative. Acyclovir was then suspended. A doppler echocardiography showed moderate left ventricular dysfunction (ejection fraction: 45%) and mild pulmonary hypertension (35 mmHg) while in milrinone (0.5 mcg/kg/min).

Seven days after admission, hemodynamics and infectious status improved. However, there was no sign of neurological improvement. The patient had no respiratory drive or cough reflex, and Ramsay scale was 6 even after sedation was removed.

**Figure 1:**
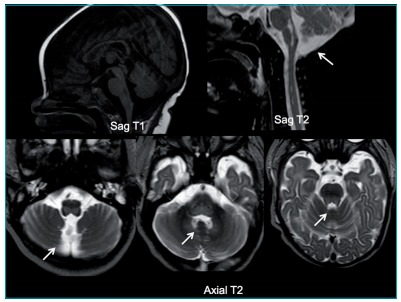
MRI: Sagittal T1, Sagittal T2 and Axial T2 views showing well-defined hypointense (T1) and hyperintense lesions (T2) at the dorsal portion of pons and bulb.

MR showed: “T1-D1 hypointense, T2/Flair hyperintense images in diffusion sequence at the dorsal medulla oblongata and pons, with involvement of VI cranial pair” (Figure 1). Hyperintense lesion was seen in the diffusion sequence, affecting the *corpus callosum*. These alterations were compatible with the Dorsal Brainstem Syndrome (DBSS), a condition occurring after hypoxic-ischemic injury in neonates and infants. The pattern of brain injury relates to the pathogenetic mechanism and provides information about outcomes and prognosis.

Two weeks after admission, vasoactive drugs started being removed, the cardiovascular function had improved, and CSF was normal. On week 3, we started weaning the patient from MV. However, the procedure was not successful and pCO_2_ would raise every time we tried to wean MV. Hence, the patient remained dependent of MV with no improvement in respiratory drive, although his neurological status improved and he started moving the limbs, doing eye contact, and reacting to painful stimuli. Tracheostomy and gastrostomy were performed at this time.

**Figure 2:**
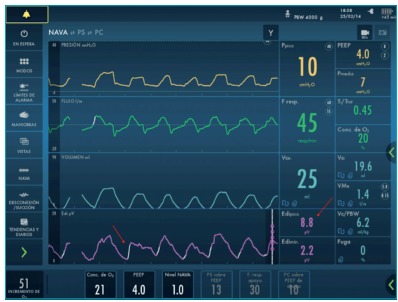
Ventilation SERVO-i with NAVA display showing Edi signal (arrows).

Since the MV weaning was challenging and the medical staff were in doubt about neural conduction integrity, we decided to use neurally-adjusted ventilatory assist (NAVA). After adequate eletrode positioning, Edi signal was detected (8 mV) and confirmed neural conduction (Figure 2). After that, a program of MV weaning using NAVA was proposed; each day the patient remained four hours in NAVA, switching to pressure support for another four hours and synchronized intermitent mandatory ventilation (SIMV) during the night. The patient was discharged from the hospital three months after admission, and is currently in homecare, receiving nebulization during the day, and bilevel positive airway pressure (BiPAP) overnight.

## DISCUSSION

DBSS is a rare entity but it should be considered whenever a neonate or infant develops a hypoxic-ischemic injury. Located in the watershed area between the paramedian and circumscribing branches of the basilar artery, the brainstem tegmentum is vulnerable to ischemic distress.^4^ According to Sugama *et al*., “lesions in this area can be seen as changes in signal intensity at MR in some cases”,^5^ ^,^ ^6^ but in other cases, they may not be visible on neuroimaging.^7^ ^,^ ^8^ These can present with or without supratentorial lesions that often involve the thalamus, basal ganglia, and periventricular white matter. MR imaging is helpful for both diagnosis and prognosis, depending on lesion extension. Prompt and adequate treatment of the shock is primordial to prevent injury expansion, promoting a better outcome for these patients. In the case reported, despite the accurate clinical assistance and readily execution of sepsis protocol, the infant developed DBSS.

NAVA is a mode of mechanical ventilation that could help determine neural conduction in DBSS patients. It is a safe method of ventilation. Most studies have shown no significant adverse events in children cared for with NAVA, and no differences in intraventricular hemorrhage or pneumothorax rates when compared to conventional ventilation.^3^ Besides that, in neural triggering, the electrical trigger coming from the brain through the vagal nerve stimulates diaphragm comcomitantly to the ventilator, therefore improving patient-ventilator synchrony, permitting breath-to-breath variability, and reducing sedation need.

We conclude that dorsal brainstem syndrome is a rare condition in infants. However, it should be considered in all young patients with hypoxic-ischemic lesions. To our knowledge, this was the first time NAVA was used to check neural conduction in an infant with this king of neurological injuries. Spreading the concept of dorsal brainstem syndrome would contribute to the recognition of similar patients and alert neurologists and pediatricians for better therapeutic approaches.
